# “Stomatitis Materna.”—The Sore Mouth of Nursing Women

**Published:** 1848-02

**Authors:** Charles B. Coventry


### BUFFALO MEDICAL JOURNAL

AND

### MONTHLY REVIEW.

VOL. 3. FEBRUARY, 1848. NO. 9.

### ORIGINAL COMMUNICATIONS.

ART. I.—"*Stomatitis Materna.*"—*The Sore Mouth of Nursing Women.* By Charles B. Coventry, M. D. *With remarks by the Editor.*

It seems not a little surprising that a disease of so severe a character, and extensive in its prevalence, has neither been named, been placed in any nosological arrangement, nor received even a cursory notice in standard works on the diseases of females. The information to be derived from books on this subject is meagre in the extreme. Its bibliography is soon told. It consists of a communication made to the Massachusetts Medical Society, by Doct. Hale, Boston, in 1838, and republished in the "American Journal of the Medical Sciences" for April, 1842. A communication to the same Journal, by Doct. Backus, of Rochester, published in January No., 1841; an article in the No. for Oct., 1842, by Doct. Shunks, of Memphis, Tennesee; and a brief commnnication from Doct. B. W. Taylor, of Florida, published in the same Journal for January 1842.

Though the disease has not been noticed by standard writers, it is neither new, nor limited in its extent. Doct. Hale speaks of its prevalence in Boston in 1830. Doct. Backus says he had met with it in practice for twenty years, and I remember that the disease was a matter of discussion with my preceptor, and other practitioners, more than twenty years since. Doct. Taylor says, that he has seen the disease in New England, in the State of New-York, in the West and in Florida. Doct. Shunk, says, at one time it was so prevalent at Memphis, that scarcely a nursing woman escaped. The frequent inquiries respecting the disease made by students from almost every section of the country, have satisfied me that, although particular localities may be exempt, its prevalence is extensive, and not confined to any particular section of the country or latitude. That the disease is not one of a trivial nature, can be testified by all who have had occasion to treat it, and by the fact that it occasionally proves fatal. How then are we to account for the neglect of standard authors to mention it? Probably it is unknown in Great Britian, as it is not mentioned either in their works or periodicals. The talented editor of the American Journal, says, that the late Prof. Dewees never met with a case, and that on inquiring of several of his medical friends in extensive practice in Philadelphia, they had never seen the disease.

*Symptoms.*—Doct. Hale, says, "it begins with a hard pimple on the edge of the tongue, generally a short distance from the tip, which is very red, and extremely painful. In many mild cases this continues a few days, and then disappears. This is the mildest form of the disease, and unless it is under the influence of medicine, does not remain in so mild a state. After a few returns, and not unfrequently a first attack, the central spot ulcerates. The ulcer is deep,. with elevated edges, surrounded by an inflamed circle, and is exquisitely painful. Several of these ulcers form on the inside of the mouth, i. e., on the tongue, and inside of the cheeks, rarely, if ever, on the gums, or palate, and, although each ulcer is of small extent, yet the inflammations around them spread over nearly the whole extent of the mouth, the salivary glands are excited, so that there is considerable salivation. The inflammation next extends to the fauces, and then to the mucus coat of the œsophagus, stomach and intestines, accompanied by diarrhœa. Doct. Backus, says: "the first accession of the disease, is sudden, often extremely so. In three hours' time after seeing your patient in health, you may find her with a scalded tongue. The first sensation of the patient referred to the tongue, is that of scalding. The color of the tongue is very peculiar, being pink, the edge and roof of the mouth having a deeper hue of this color, accompanied with a profuse watery discharge from the mouth, extremely hot, so much so as to give a scalding sensation to the face when passing over it. The appetite is usually good, often ravenous, but only the blandest food can be taken into the mouth without producing the most intense pain. After a continuance of this state of the mouth for a few weeks, there appears slight ulcerations on the end, or edges of the tongue, or about the fauces. Sometimes the disease commences with slight ulcerations, and this scalding tongue and fauces follows. The bowels are constipated, or soon become so, no fever is present, but often excessive irritations of the whole system, in consequence probably, of the want of rest, as the continued pain of the fauces, together with the excessive, and continued flow of burning saliva, prevents any comfortable rest, night or day; tongue always free from any coat." Doct. Shunks, says: "in most of the cases in which this form of sore mouth occurs after confinement, the last months of gestation are attended with functional derangement of the liver, stomach, and bowels, evinced by acidity, flatulency, white viscid secretions, producing vomiting, which, instead of occurring in the morning, as in the early months of pregnancy, mostly takes place soon after laying down at night. The symptoms, when distressing, are attended with an irregular, often morbidly craving appetite, and constipated bowels. If not relieved, the tongue becomes red, the papilla flaccid, and projecting, giving it a peculiar rough and raw appearance, with diffused redness, and tenderness of all the inside of the mouth and throat. The secretion from the mouth is acrid, producing, a scalding, or burning sensation." The irregularity of the bowels and previous constipation, I believe are seldom absent, though, probably not essential to the disease; they, however, are seldom noticed by the patient, the sore mouth being the first thing to attract attention. The soreness of the mouth often comes on suddenly, sometimes, as described by Doct. Hale, with elevated and hardened pimples at other times with a general scalding, as described by Dr. Backus, and in others with a combination of both. The soreness of the mouth will sometimes disappear as suddenly as it came on, but is always liable to return in consequence of errors in diet. In a patient who has suffered from it, it is almost certainly reproduced by a meal of indigestable food. If the irritation extends to the stomach and intestines, diarrhœa supervenes, or the patient is troubled with alternate diarrhœa and constipation. When the diarrhœa commences, the soreness of the mouth usually somewhat abates ; the tongue is perfectly smooth, the pink color becoming gradually lighter as the patient becomes exhausted; all desire for food is destroyed, and if much is taken, it is either rejected by vomiting, or the diarrhœa is agravated, and the exhaustion increased. The patient's strength is rapidly exhausted, particularly if she continues to nurse the child. If no local disease supervene to destroy the patient, the strength gradually fails from deficient nourishment of the system; effusions of water into the cellular system, and the different cavities, take place, and she dies, as it were, from exhaustion. Snch, at least, has been the result of the only fatal cases which I have seen. I know of no instance where an examination was made after death.

While engaged in writing the above, I was called to see Mrs. R. with the disease. Mrs. R. was confined about two weeks since ; suffered somewhat from hæmorrhage, but, in other respects, the labor was natural and easy; has suffered from sore mouth with two former children, but much less the last confinement; health tolerable previous to confinement, but bowels costive, although now irregular; most of the time constipated, sometimes attacks of diarrhœa; pulse feeble, but no excitement; appetite good; tongue very slightly furred, edges red, with several small hardened elevations; says the soreness is very irregular, sometimes in the morning it feels almost well, and before night may be as bad as ever; some redness of the throat and fauces.

*Cause.*—Although it is thought by all those who have written on the subject, that there is an intimate connection between the disease and the process of nursing, yet there must be some other cause operating in connection, in order to produce it. Doct. Backus, says: "no man ever had the disease, or ever will have it." Doct. Hale says: "that it is confined to nursing women, if we except some few cases of its recurrence during pregnancy in those who have previously had the disease." Doct. Taylor, says: "In an exten sive practice of seven years, I have never met with a single case of sore mouth in man, or in woman who did not give suck, which was in the least analogous to the species of sore mouth under consideration." Notwithstanding these statements, the disease is by no means unfrequent in pregnant women, or women previous to confinement. I have frequently known it to harrass them during nearly the whole of their pregnancy; sometimes getting better, when some imprudence in diet would agravate the disease. In one case it seemed to alternate with irritation of the stomach, and the two almost exhausted the patient previous to confinement. All admit that recovery is facilitated very much by weaning the child, and that in many cases it is indispensable to the recovery.

It is, however, evident that other causes must co-operate in the production of the disease. The only writer I have seen who has attempted to assign this additional cause, is Doct. Shunks, of Memphis, Tennessee. He says: "The predisposing cause of this disease seems to be marsh miasm, combined with humidity of the atmosphere, as the disease never prevails in high, dry regions of the country, free from humidity, and marsh miasm. In further confirmation of this opinion, deranged secretion of the stomach and liver are found invariably to precede and attend the disease. As the country has been cleared, improved, and made dryer, and, consequently, the humidity and the miasmata in the atmosphere diminished, the endemic fever of the country has become less prevalent, and this endemic sore mouth has diminished in its prevalence and severity in proportion. Formerly, at all seasons of the year, few women escaped it, in some degree, during lactation; now, since the country has become more exempt from intermittents, this affection has become more rare, attacking those, mainly, who are unacclimated, or of feeble health. That the predisposition is thus produced is farther evinced by the fact, that chronic diarrhœa, in humid, miasmatic localities, in males, presents many of the general symptoms of this disease peculiar to nursing females." In this city and county, cases of diarrhœa, of a protracted and chronic character, present themselves, especially in persons of intemperate habits, accompanied with sore mouth, and a peculiar red and raw tongue, with ulceration of its under side, and inside of the mouth. These cases are always produced, and accompanied by morbid secretions of the liver and stomach, but are different from the affection in females in the characteristic symptoms." Further observation is necessary to establish, conclusively, the theory, or views of Doct. Shunks, but in so far as our present knowledge extends, it goes to confirm his opinion. In the city of Utica, 20 years since, the fevers supposed to be produced by marsh miasm occurred only in persons who had been exposed to the cause in some other part of the country. The disease under consideration, if it ever occurred was very rare. Subsequently, both Intermittent and Remittent fevers, as well as this peculiar sore mouth, have become frequent, and although some cases occur every season, I think I have noticed them as most common in such seasons as the cases of fever have prevailed. The deranged condition of the digestive organs, the constipation, and morbid secretions from the liver, seem to point to a similar cause. This affection, in addition, seems to obey the laws which govern other endemic diseases, being much more prevalent some seasons than others, being found to prevail in certain localities, whilst neighboring towns are exempt. That the particular state of the system, in pregnancy should modify the operation of certain causes, so as to produce an affection which seems peculiar to women in that particular condition, is by no means surprising. We have a striking illustration in the malignant forms of puerperal fever. The latter was long considered a specific disease, to which puerperal patients are alone subject. I believe it is now demonstrated that it arises from the same cause that produces other forms of malignant fever, as typhus and malignant erysipelas, being modified by the particular state of the system. The treatment, as we shall endeavor to show, which has been found most successful, seems to indicate a similar origin.

*Treatment.*—All the writers I have met with, admit that the sore mouth is only a symptom of a more general derangement, though this may be the most prominent and most distressing symptom, and consequently, that remedies applied to the mouth, as washes, &c., have little effect, except in alleviating the severity of suffering, exerting but little control over the disease. Doct. Hale says:—"in the treatment of this affection, local remedies are of very little service. In general, mouth washes, and gargles avail so little as to give scarcely a momentary relief." Doct. Backus says: "I have, for the most part, treated the disease as arising from a vitiated secretion of the stomach, produced in some unknown manner, by the process of lactation, although, in a great many cases, I have had no reason to believe the stomach much disordered, for the patient, three hours before, might have been perfectly well. I formerly gave emetics, cathartics of calomel, or mild ones of blue pill, with local applications to the mouth, but this treatment often failed, the disease, though mitigated for the moment, still went on more and more severely. I have used the bismuth, and other metallic tonics, without effect. About five years since, I adopted a new method, which I have found more efficacious than any I had before pursued, As soon as the symptoms appeared, I made a free and continued use of the following pill:—℞. carb. ferri, grs. lxv.; rhei and G. aloes a. a. grs. xxiv.; pulv. ipecac and Castile soap, of each, grs. xii.; mix and divide into fifty pills; two to be taken two or three times a day, or often enough to keep the bowels open. I have always observed that an artificial diarrhœa occasioned by them usually afforded almost immediate relief, and often during the continuance of the disease, a spontaneous diarrhœa, from any cause, relieves the mouth at once. These pills, accompanied with an astringent wash of blackberry, or marsh-rosemary root, or a solution of nitrate of silver, seldom fail to afford relief, but, latterly, some cases have resisted this, and every other mode of treatment, and I have had to wean the child, when the disease was cured at once, and did not return." Doct. Hale says: "in any stage of the disease, if the child is taken from the breast, the affection of the mouth heals with great rapidity. He adds: "the same is true. in the earlier part of the disease, of the diarrhœa, and other sympathetic affections, and, indeed, it generally is so when the weaning takes place at any period." Doct. Hale observes, "that it not unfrequently happens that the stomach is disordered, though this is not essential to the disease, and when it so, emetics are necessary. The emetics are but preparatory to the direct treatment of the disease and there are many cases in which it may be dispensed with. For the cure, the chief reliance must be upon tonics, those, particularly which give vigor and action to the stomach, with little general excitement." Doct. H. recommends cinchona infused in lime water, carbonic acid in different forms; also, porter, beer, &c. He says: "I have known some cases in which calomel, combined with opium has been given with advantage, though he had not himself met with any which seemed to call for it. Doct. Shunks, says: "in the more robust and plethoric, the feverish excitement must be relieved by bleeding, which should be followed by alteratives and laxatives, such as blue mass, calomel, magnesia, rhubarb, &c., in small doses, with plain diet. In those of more feeble health, in whom little or no feverish excitement exists, a combination of blue mass, carb. iron, ipecac, rhubarb and aloes, in such proportions as suit the particular case, answers well as an alterative, and tonic laxative. Ipecac, alone, in doses of from one half to two grs, is a good remedy. Doct. Christison, the oldest practitioner in this city, relies mainly on ipecac, in doses of from half to two grains, repeated three or four times a day. Others prefer blue mass, carb. of iron, rhubarb, and sometimes opium, combined with ipecac; or blue mass, and soda with the drink. Active purgatives of all kinds are injurious. As a wash for the mouth, the infusion of blood root is perhaps the best. In bad cases, however, when there is much debility, emaciation, and nervous irritation of the system, the experience of all the physicians in this region of country concurs in the indispensable necessity of weaning the child to cure the disease, and in some cases, to save the life of the patient. The improvement from an alarming state of debility, and emaciation, after weaning the child, in often prompt and rapid."

Doct. Taylor, states, that after having tried various tonics, vegetable and mineral, and laxatives, with only partial success: "I have found that equal parts of flower of sulphur and cream of tartar, administered in broken doses, two or three times a day, so as to keep the bowels in a soluble state, constitutes the best treatment, as regards internal remedies. The combination of creams of tartar and sulphur, seems to have almost a specific effect over this disease The best eternal application, I think, is borax, either in the form of solution sweetened with honey, or loaf sugar." A highly intelligent practitioner of my acquaintance has informed me that he was in the habit of using the nitrate of silver, both internally, and as a wash for the mouth, in this disease with success. Others have assured me they have seen the blood root, given internally, with advantage, and several students of medicine have told me that their preceptors considered the cubebs as almost specific.

Previous to the publication of Doct. Backus, I was in the habit of giving a combination of phos. iron, calumba, and carb soda, after endeavoring to correct the secretions, when they seemed deranged, by the use of the blue pill, or small doses of calomel, combined with some laxative. This answered a useful purpose, the greatest objection arising from the difficulty of taking it on account of the bulk, and its sometimes proving offensive to the stomach. Latterly I have used the pill of Doct. Backus as a substitute, and have been much pleased with the effect. If used early in the disease, with a carefully regulated diet, it will correct the deranged condition of the system, and remove the disease. I have, however, sometimes found it necessary to wean the child, and, in some few cases, the disease has continued even after the child was weaned. My own observations and experience in the disease, which have been considerable, would convince me that it was by no means so exclusively confined to nursing females as the writers quoted represent. Whenever it prevails among nursing women, it is frequently found in those who are pregnant, before confinement. Although weaning the child has usually a favorable influence upon the disease, and is often indispensable, it is not so uniformly successful as we would be led to hope from what has been written, the disease sometimes continuing, notwithstanding this, and every other known means. In the most obstinate case with which I ever met in my own practice, the patient was very much exhausted before her confinement, the child was taken at once from the breast, there was great prostration of strength, an entire loss of appetite, bowels constipated unless moved by medicine, but the mildest cathartics would bring on diarrhœa ; if food to any amount was taken, it was usually rejected, and if not, it produced flatulency, and pain in the stomach and bowels. After the failure of all the ordinary means, my patient was apparently benefited by, or at least began to improve under the use of a combination of minute doses of calomel, ipecac, and sul. of morphine, with the repeated application of blisters over the region of the stomach, and the occasional exhibition of a mild enema to move the bowels, the food being limited to the smallest quantity and mildest kind, My own experience, then, would concur with the almost unanimous testimony of those who have written upon the subject, that the most successful treatment in the early stage consists in a combination of tonics with mild laxatives, continued for a considerable time. When the stomach is much deranged at the commencement, a mild emetic may be useful; and when the appearance of the discharges indicate a deranged secretion from the liver, the addition of minute doses of calomel, or blue pill, avoiding salivation. I think the mineral tonics preferable, though I have given both the sulphate of quinine and calumba with advantage.

I am indebted to Doct. J. Z. Havens, of Utica, for the suggestion that the congress water combines the desired properties, viz., of a laxative, and mild tonic, together with the carbonic acid (which has frequently been recommended) in a convenient and palatable form. I have since given it myself, and seen it administered by others with decided advantage. In those troublesome cases attended with great irritability of the stomach, it will be found particularly valuable, often setting well on the stomach, when almost every other form of medicine is rejected. As a local application, an infusion of the sanguinaria canadensis, or a solution of nitrate of silver, or borate of soda, or any of the mild astringent infusions, may be used. Throughout the whole treatment the utmost caution should be used with regard to the diet. I have frequently known a relapse occur from the patient imprudently indulging in a single meal of improper food.

Should the means recommended, or others which may be adopted, not readily succeed in arresting the disease, I believe it would be best, at once to wean the child, or procure another nurse. If the disease has extended from the mouth to the stomach and intestines, producing diarrhœa, the child should be immediately weaned, the patient confined to the simplest food, and counter irritation produced over the region of the stomach. Should the disposition to diarrhœa continue, small doses of morphine, or some other preparation of opium, may be necessary to control the discharges. Great caution should be used in the administration of active cathartics, as they exhaust the system very rapidly. If it should be necessary to relieve constipation, it should be done by the use of mild enemas, in preference to cathartics by the mouth.

*Remarks by the Editor.*—As the subject of the foregoing valuable communication is one of much practical importance, and, as the writer justly remarks, has received very little attention from systematic authors, we are led to subjoin, briefly, the views of the pathology and treatment which have resulted from our own experience and reflections. It is indeed surprising, as our correspondent observes, that an affection of so important a character, is, as yet, without a classical title, The name which prefixes the article, *stomatitis materna*, is one which is, so far as we know, original with ourselves. Its appropriateness is based on the fact that the affection is limited to nursing women, or mothers. In the absence of any title which has been generally adopted, we venture to submit this.

What is the pathology of the affection? We regard it as dependent upon anœmia, induced by lactation. The blood becomes impoverished by the continued drain upon its nutritive elements to form the lacteal secretion, and this is one of the local consequences which occasionally follow. In all cases that have fallen under our observation patients present unequivocal evidences of the anœmic state, and in proportion as this state has been relieved by appropriate measures, the affection of the mouth has improved. Why this local disorder should ensue as a result of anœmia we cannot tell, more than we can explain why various other local disorders are attributable to the same morbid condition. Still less can we understand why this particular local disorder should be characteristic of the anœmia induced by lactation or pregnancy, and not often, if ever, occur as a consequence of anœmia induced by other causes. In other words the fact that this form of stomatitis is peculiar to nursing women, in the present condition of science, is inexplicable. Doubtless there exists some special connection between the disease and the kind of impoverishment which the blood undergoes in consequence of the withdrawal from this fluid of the elements which go to form the lacteal secretion; but our knowedge of chemico-vital chemistry does not suffice to trace the circumstances which constitute this special connection.

This is by no means the only instance in which the mucus membrane takes on disease as a result of prior deterioration of the fluids. We may instance the stomatitis which occurs in the last stages of pulmo-tuberculosis, and other chronic diseases; softening of the mucus tissue of the stomach and intestines from inanition; scrofulous ophthalmia; perhaps we may add the follicular affection of typhoid fever, &c.

That the affection has seemed to arise from miasmata is explicable, when we consider that to the latter alone anœmia is often referible, and consequently the effect of lactation is increased by conjunction with miasmatic influences. That it is wholly due to miasm is, however, sufficiently disproved by the fact that it occurs in localities where miasmatic emanations are unknown.

If this view of the pathology be correct, the proper therapeutical principles are readily deducible. The seat of the disease is not in the mouth, but it is a blood disease. Hence, we should expect, what experience declares to be the case, that collutories are of little or no avail. To reach the *fons et origo* of the difficulty, the blood must be restored to a healthy condition.

The first question with the practitioner is, can this be accomplished while the remote cause of the disease (lactation) continues. Our own experience teaches us that in the large proportion of cases weaning becomes necessary. This affection frequently does not occur until the blood has undergone deterioration to such extent that restoration is impossible without removing the remote cause. Whenever the patient is much prostrated, and, especially, if diarrhœa co-exists, the lacteal secretion should be allowed to cease without delay.

In most cases, however, it will be proper to give remedies a fair trial before deciding on the necessity of weaning. And, if we mistake not, the success of the treatment dictated by the foregoing view of its pathology, will, in not a few instances, strikingly exemplify the correctness of the latter. The treatment is that called for by the anœmic state, viz: nutritious diet, especially animal food and ferruginous tonics. Should the bowels be costive, or any functions be disordered, they will claim appropriate measures, but the grand object is to improve the powers of assimilation, and thereby restore the healthy condition of the blood. A great difficulty with a view to this object arises from the inability of the patient to take proper food in proper quantities, owing to the tenderness of the mouth. We have known patients who constantly suffered from hunger, which they would endure, rather than incur the pain incident to satisfying fully the appetite. Local applications should be tried, and, by alleviating the soreness, they may conduce to permanent benefit, by permitting the patient to take more nourishment. To the local remedies which are mentioned in the foregoing article, we would add, a solution of tannin in port wine, used as a collutory, of sufficient strength to cause a decidedly astringent effect, without occasioning much pain. We have employed this remedy, of late, in some cases, with advantage.

Of the tonics to be prescribed, there are various preparations which are appropriate, and the peculiarities of different cases will probably be suited by different forms. The citrate of iron, in combination with quinia, given in concentrated solution, as suggested by Dr. Meigs, has been a favorite prescription with us, and we know of no form more generally applicable.

Exercise in the open air appears to us one of the most important elements in the treatment.

Frequently the child may be partially weaned. Some mothers are accustomed to nurse frequently, or almost constantly during the night time, when loss of sleep is to be added to the cause of the disease which is more direct in its operation. This foolish custom should be interdicted.

Should these measures fail, weaning should be directed, and, in nearly all cases, the disease rapidly subsides so soon as this is done.

We give the foregoing views, not because we suppose them to be entitled to any merit of originality, but for the reason which has led our esteemed friend to submit his able article, viz: that the subject is one of practical importance, and one which has been surprisingly overlooked, or neglected by systematic writers.

